# Cognitive function and heart rate variability in open and closed skill sports

**DOI:** 10.1080/07853890.2023.2267588

**Published:** 2023-10-12

**Authors:** Sandipana Chakraborty, Chinmay A. Suryavanshi, Kirtana R. Nayak

**Affiliations:** Department of Physiology, Kasturba Medical College Manipal, Manipal Academy of Higher Education, Manipal, India

**Keywords:** Attention, basketball, cognition, heart rate variability, swimming

## Abstract

**Objectives:**

The differences in sporting environments between open and closed skill sports impose unique demands on athletes’ cognitive and motor capabilities. Our study aims to investigate and compare cognitive function and Heart Rate Variability (HRV) among individuals involved in different sports, namely basketball, swimming, and a sedentary non-sports group.

**Materials and methods:**

The study consisted of three groups, namely basketball players, swimmers, and sedentary individuals, with each group comprising twenty-six participants. HRV was assessed with the help of PowerLab. Cognition was assessed using the Ebbinghaus Memory Procedure Test (EMT), Go/No-Go Task (GNG), Color Stroop task, Trail Making Test (TMT), and Letter Cancellation test (LCT).

**Results:**

The results of the Multivariate Analysis of Covariance (MANCOVA) analyses indicated that there was significance between the groups. However, no significant differences were observed between swimmers and basketball players in cognitive functions and HRV measures. Overall, the sport group outperformed the sedentary group. Specifically, basketball players and swimmers completed LCT and TMT faster than the sedentary group (*p* = 0.044 and *p* < 0.001 for basketball players, *p* = 0.002 and *p* = 0.001 for swimmers). Additionally, basketball players took fewer trials in EMT (*p* = 0.013) and less time (*p* = 0.026) compared to the sedentary group.

**Conclusion:**

The results of the study indicate that sports training, regardless of sport type, positively impacts overall cognitive function. However, no significant differences were observed in cognitive task performance and HRV measures between open and closed skill sport players. These findings suggest that sports can enhance cognitive functions, regardless of the sport played.

## Introduction

Sports can be categorized into two types: open skill and closed skill sports, which are differentiated by the impact of the environment on sporting skills [1]. Sports in which players are required to react in an unpredictable, dynamic, and externally paced environment are called open skill sports. Open skill sports entail the ability to react in a rapidly shifting, unpredictable, and externally paced environment, such as basketball, tennis, and fencing. In contrast, closed skill sports involve a comparatively steady and self-directed sporting environment, such as running, swimming, and track and field events. Basketball is an open skill sport where players must make tactical decisions, high-intensity movements, abrupt directional changes over short distances, and specific whole-body movements in response to the continuously changing sporting environment while attacking the opposing team’s hoop [1–3]. In contrast, swimming is classified as a closed skill sport that involves a stable and self-paced sporting environment, as in track and field events [4]. In closed skill sports, the movements performed by athletes tend to be quite fixed, with consistent environmental and sport-specific demands persisting throughout the entirety of the action’s execution [5]. These differences in sporting environments place distinct demands on athletes’ cognitive abilities. Closed skill sports typically adhere to predetermined patterns and are generally regarded as having reduced cognitive demands [6]. Additionally, most sports require higher psychological and physical endurance standards to counter the dynamic sporting environment [7]. So, to excel in such sports, an athlete requires compatible motor and sensory cognitive skills [8].

Hillman et al. have documented the beneficial effects of exercise and competitive sports training on cognitive and brain function, which can be observed at the molecular, cellular, and behavioural levels by developing more efficient brain networks [9]. Studies support the notions that physical activity not only reduces cognitive decline but also leads to improved cognition in older adults, that individuals who engaged in higher levels of physical activity at baseline had a 38% reduced risk of cognitive decline at follow-up compared to those who led a sedentary lifestyle at baseline [10]. Aerobic fitness & sports training have been demonstrated to enhance cognitive functions like executive control, conflict solving, and inhibitory control [11]. Inhibitory control, which involves the ability to suppress inappropriate actions in a given situation, plays a crucial role in choosing appropriate behaviours in daily life and is particularly relevant to successful sports performance. To assess inhibitory control, previous studies have utilized the Go/No-go task, which has consistently demonstrated that athletes, such as basketball players and fencers, exhibit significantly fewer errors and faster response times than non-athletes [12]. Both reaction time (RT) and anticipatory skills are critical in an athlete’s perceptual abilities. In open skill sports such as basketball, anticipatory skills play a crucial role in decision-making processes, as players need to rapidly gather relevant information from their surroundings to optimize decision-making time. This enables them to allocate more time for executing specific motor behaviours [13]. It has been observed that athletes from open skill sports are more pliable in executive functions, decision-making, and visual attention [14]. According to a recent systematic review, the current evidence leans towards open skill sport being more effective in enhancing certain aspects of cognitive function compared to closed skill sport. However, it is still too early to determine the full impact of open skill versus closed skill sport on a particular cognitive domain [6]. In line with this research direction, the current study examines the variability in cognitive function among athletes and non-athletes, specifically comparing basketball players and swimmers.

The maximal capacity for oxygen transport and utilization during progressive exercise (VO_2_ max) is a valuable marker of cardiorespiratory fitness and endurance. Heart Rate Variability (HRV) is a crucial indicator for illustrating the modulation of cardiac activity by the autonomic nervous system [15]. Engaging in regular physical activity and participating in sports training at varying intensities are frequently linked to an elevation in cardiac vagal tone. A study conducted by Bherer et al. proposes an elevation in higher-order cognitive function resulting from improved cardiorespiratory functions induced by aerobic training [16]. The Neurovisceral Integration model postulates the existence of a neuroanatomical linkage between the autonomic and central nervous systems known as the central autonomous network (CAN) [17]. This model proposes an interplay between executive function processes controlled by the prefrontal cortex (PFC) and parasympathetic cardiac regulation. Cognitive functions such as sustained attention, behavioural inhibition, and working memory have been linked to the PFC. Suess et al. have observed enhanced cardiac parasympathetic control in individuals exhibiting superior performance in cognitive tasks [18]. A study employing a working memory task unveiled that individuals with high HRV demonstrated enhanced and consistent performance, irrespective of environmental stress [19]. Moreover, studies conducted by Lane RD et al. utilizing pharmacological and neuroimaging methods provided evidence supporting the association between prefrontal cortical activities and cardiac vagal functions [20,21]. Generally, cardiovascular arousal tends to enhance motor actions. It can be argued that executive cognitive processes, particularly those related to action selection, preparation, and cognitive effort, influence autonomic balance by shifting the sympathovagal balance towards states of heightened cardiorespiratory arousal conducive to action readiness [22]. While previous studies have highlighted athletes’ superiority in HRV measures and cognitive abilities, it remains unclear whether these factors can be regulated differently across different sport types due to the distinct cognitive and motor demands associated with each sport.

Based on previous models and studies, HRV has been established as a non-invasive method for determining CAN activity and executive functioning [15,17,23]. According to a recent systematic review, there is evidence that vagally mediated heart rate variability can be used to predict cognitive inhibition and flexibility [24]. Hence, we propose that the improved executive performance observed in highly fit individuals is linked to HRV, which influences prefrontal neural functions. Our objective is to investigate and compare cognitive function and HRV among individuals engaged in different sports, specifically basketball and swimming, and a sedentary non-sports group.

## Methods

In this study, a total of 78 individuals ranging in age from 18 to 30 years were included. Participants were divided into three groups: 26 basketball players, 26 swimmers, and 26 sedentary group controls. The inclusion criteria for the basketball players and swimmers required regular participation in their respective sport for at least 3 h per week for six months. The sedentary group control participants were individuals who did not engage in any regular sports activity. Participants with a history of cardiovascular disease, diabetes, preexisting injuries, neurological problems, hearing or vision problems, or smoking were excluded from the study. All participants provided written informed consent before taking part in the study.

The study was conducted in a quiet room within the Physiology department, with regulated temperature, during the morning hours. Participants were instructed to avoid strenuous physical activity and the consumption of caffeinated food or drinks for a day prior to the examination. The physical activity level of the participants was assessed using the ‘Short last seven days self-administered’ format of the International Physical Activity Questionnaire (IPAQ). Following this, the participants underwent cognitive assessments followed by measurement of HRV.

### Aerobic fitness assessment

Fitness level and VO_2_ max were assessed using the Cooper 12-Minute Run Test [25]. This test was performed according to the protocol developed by Dr. Kenneth H. Cooper, where participants were instructed to run as quickly as possible for 12 min at a steady pace between two points of a certain distance, and the total distance covered was measured. The estimated VO_2_ max was calculated based on the distance covered, following the procedure outlined in the original protocol [25].

HRV was recorded and analyzed according to the published standard guidelines using the Powerlab data acquisition system (PowerLab 4/25 T and Chart v5.4 Pro, AD Instruments, NZ) [26]. HRV was recorded with the participant lying in a supine position in a quiet room for 12 min. Subsequently, an artefact-free segment of 300 s of recording was selected for analysis. HRV data were analyzed for the time, frequency, and Poincare Plot.

### Cognitive tests

Ebbinghaus Memory Procedure Test (EMT), Go/No-Go Task (GNG), and Colour Stroop task were assessed using Psychology Experiment Building Language (PEBL) 2.0 software [27]. Participants were provided with two sets of sheets for the Trail Making Test (TMT). The first set comprised exclusively numbers, whereas the second set contained a combination of numbers and alphabets. During the initial test phase, participants were instructed to connect the numbers from lowest to highest (1–25) sequentially. In the subsequent phase, participants were instructed to alternate between numbers and alphabets, creating a sequence (1-A-2-B-3-C). Participants were instructed to refrain from lifting their hands until they completed the entire sequence. The total duration required to complete the test and any errors encountered were accurately recorded. Each Letter Cancellation test (LCT) consisted of 960 letters, with 25% designated as target letters. Participants were instructed to strike out the target letters using a pencil. The total time taken to complete the test and the number of errors and omissions were noted.

### Statistical analysis

Data from all the study groups were analyzed utilizing SPSS, version 16. Descriptive statistics, encompassing mean and standard deviations, were computed. ANOVA was employed to compare quantitative variables. The MANCOVA test was employed to evaluate the impact of sport type on cognitive tasks and HRV tasks. The association between various parameters was assessed using Spearman correlation analysis. The significance level for all analyses was set at *p* < 0.05.

## Results

In this investigation, a total cohort of 78 participants were enrolled as subjects, comprising 37 males and 41 females. Demographic information regarding the participants, as well as their levels of physical activity and VO_2_ max, are detailed in Table 1.

**Table 1.** Demographic & physiological data of the participants.

Variables	Sedentary group	Basketball players	Swimmers
Number of participants	26 (10 Males, 16 Females)	26 (14 Males,12 Females)	26 (13 Males,13 Females)
Age (year)	21.5 + 3.0	20.4 + 2.5	20.2 + 2.8
Height (cm)	166.35 ± 10.46	170.69 ± 11.72	165.12 ± 7.74
Weight (Kg)	66.24 ± 16.15	65.31 ± 11.24	64.60 ± 9.37
BMI (Kg/m^2^)	23.71 ± 4.13	22.32 ± 2.56	23.72 ± 3.16
IPAQ scoring	972.81 + 528.51	4688.78 + 1776.08	3103.11 + 1551.67
VO_2_ max (ml/min/kg)	31.34 + 7.07	56.30 + 8.69*^	48.44 + 10.88*

Data are mean ± SD.

IPAQ: International physical activity questionnaire. *Significantly different (*p* < 0.05) from sedentary group (*p* < 0.05); ^significantly different (*p* < 0.05) from swimmers’ group (*p* < 0.05).

Examining participant descriptive data across the three distinct groups was executed through rigorous comparative analyses. ANOVA was employed to discern any notable differences among the groups for height [F (2, 75) = 2.17, *p* = 0.121], weight [F (2, 75) = 0.11, *p* = 0.894], and BMI [F (2, 75) = 1.48, *p* = 0.233]. These analyses demonstrated no statistically significant disparities across the groups regarding height, weight, or Body mass index (BMI).

However, a noteworthy contrast emerged when evaluating VO_2_ max, with a pronounced discrepancy observed [F (2, 75) = 52.09, (*p* < 0.001)]. Subsequent post-hoc Bonferroni tests were conducted to unravel the specific nature of these distinctions. These analyses elucidated that both Basketball players (*p* < 0.001) and Swimmers (*p* < 0.001) exhibited significantly elevated VO_2_ max in comparison to the sedentary group. Furthermore, it was discerned that basketball players demonstrated a higher VO_2_ max than swimmers (*p* = 0.007) (Table 1).

## Analysis for cognitive tests

To test whether each sports mode has different impacts on cognitive functions, MANCOVA was performed in which sport type served as the independent variable (swimmers, basketball players and non-sports sedentary group) and the cognitive function measures served as the dependent variables, whilst controlling for VO_2_ max and BMI (Table 2).

**Table 2.** Cognitive function test parameters in the three groups.

	Sedentary group	Basketball players	Swimmers	F(2,73)
No of errors in LCT	16.96 + 11.52	7.96 + 2.93^a^	12.58 + 8.07	7.56*
Time LCT (seconds)	559.69 + 124.02	485.85 + 89.09^a^	455.73 + 103.74^a^	6.25*
Total time in TMT (seconds)	97.15 + 26.77	61.08 + 13.96^a^	68.12 + 13.81^a^	23.72**
Trials for EMT	18.73 + 7.86	14.04 + 4.21^a^	15.12 + 4.47	4.73*
Time taken for EMT (milliseconds)	596.45 + 259.72	446.89 + 143.59^a^	467.39 + 190.29	4.16*
Mean accuracy GNG (%)	96.29 + 2.61	96.69 + 1.82	96.49 + 2.37	0.19
Mean reaction time GNG (milliseconds)	598.17 + 97.18	567.40 + 62.56	569.73 + 84.70	0.86
Total errors in stroop task	10.38 + 8.52	7.73 + 5.92	8.19 + 5.96	0.80
Mean reaction time in stroop task (milliseconds)	863.62 + 171.42	797.50 + 144.60	810.49 + 142.94	0.94

Data are mean ± SD. MANCOVA results of differences across three groups while controlling for autonomic function parameters. *p < 0.05; **p < 0.001. *Post-hoc* analysis indicated a group; ^a^significantly different from sedentary group. LCT: letter cancellation task; TMT: trail making task; EMT: Ebbinghaus memory procedure test; GNG: Go/No Go task.

The results of the MANCOVA are presented in Table 3. Findings showed a statistically significant main effect of sport type on the combined dependent variables after controlling for VO_2_ max and BMI, *F* (18, 132) = 2.21, *p* = 0.007; Wilk’s *Λ* = 0.59, partial *η*^2^ = 0.235. However, this effect may be mediated through the autonomic nervous system rather than differences in sport types. In order to assess the distinct effects of various sports, a MANCOVA analysis was conducted using covariates of a measure of heart rate variability (HF and LF/HF). The results revealed a significant main effect of sport type on the dependent variables when considering the influence of HF and LF/HF, *F* (18, 130) = 3.71, *p* < 0.001; Wilk’s *Λ* = 0.44, partial *η*^2^ = 0.34.

**Table 3.** Heart rate variability measures in the three participant groups.

	Sedentary group	Basketball players	Swimmers
Heart rate (bpm)	73.96 + 8.98	67.73 + 10.42	65.81 + 10.62
SDRR (ms)	54.65 + 17.97	62.85 + 24.17	77.31 + 30.28
RMSSD (ms)	50.58 + 20.78	60.35 + 36.11	78.58 + 44.62
LF (ms2)	1040.12 + 1579.89	1031.50 + 792.57	1822.77 + 1735.76
HF (ms2)	1347.46 + 1005.82	1680.38 + 1531.63	1934.77 + 1720.75
LF/HF	0.73 + 0.72	0.92 + 1.16	0.92 + 1.14
LF nu	37.27 + 16.25	40.23 + 17.52	39.19 + 17.08
HF nu	61.09 + 16.23	57.08 + 16.77	58.46 + 16.89
SD1	35.81 + 14.70	42.85 + 25.71	55.42 + 31.32
SD2	68.04 + 22.46	76.65 + 26.45	92.42 + 30.00

Data are mean ± SD.

HR (bpm): resting heart rate in beats per minute (bpm); SDRR: standard deviation of all R-R interval; RMSSD: root mean square difference of successive R-R interval; HF: high frequency (HF; 0.15–0.4 Hz); LF: low frequency (LF; 0.04–0.15 Hz); SD1: standard descriptor 1; SD2: standard descriptor 2; nu: normalized unit; ms: millisecond; ms2: milliseconds squared. *Significantly different (*p* < 0.05) from sedentary group.

Univariate analyses showed that sport type had significant effect on the number of errors in LCT, [*F*(2, 73) = 7.561, *p* = 0.001, partial *η*^2^ = 0172], time taken to complete LCT task, [*F*(2, 73) = 6.252, *p* = 0.003, partial *η*^2^ = 0.146], total time required for TMT task, [*F*(2, 73) = 23.721, *p* < 0.001, partial *η*^2^ = 0.394], Trials required for EMT, [*F*(2, 73) = 4.735, *p* = 0.012, partial *η*^2^ = 0.115] and time required for EMT task [*F*(2, 73) = 4.168, *p* = 0.019, partial *η*^2^ = 0.102]. However there were no differences in accuracy scores of GNG task, [*F* (2, 73) = 0.198, *p* = 0.821, partial *η*^2^ = 0.005], mean reaction time in GNG task,[*F*(2, 73) = 0.863, *p* = 0.426, partial *η*^2^ = 0.0230] and total errors in ST [*F*(2, 73) = 0.806, *p* = 0.451, partial *η*^2^ = 0.022] and reaction time in ST [*F*(2, 73) = 0.940, *p* = 0.395, partial *η*^2^ = 0.025].

Following the initial analyses, post-hoc comparisons employing the Bonferroni test were conducted. Comparing basketball players to swimmers revealed no statistically significant differences in cognitive task performance. However, as anticipated, a notable difference was observed when contrasting the performance of the sports group with that of the sedentary group. The sports group individuals demonstrated significantly better cognitive task performance (Figure 1).

In the context of the LCT, *post-hoc* Bonferroni tests unveiled that basketball players exhibited a diminished number of errors (*p* < 0.001) and displayed expedited task completion compared to their sedentary counterparts (*p* = 0.044). Similarly, swimmers exhibited a swifter task completion time in contrast to the sedentary group (*p* = 0.002).

In the TMT task, both basketball players (*p* < 0.001) and swimmers (*p* = 0.002) demonstrated a reduced task completion time when compared with the sedentary group.

In the EMT task, basketball players showcased a significantly diminished number of trials required for task completion (*p* = 0.013) and a shorter task completion time (*p* = 0.026) than the sedentary group.

## Analysis for HRV

For assessing whether each sports type has different impacts on the autonomic nervous system, MANCOVA was performed in which sports type served as the independent variable (swimmers, basketball players and non-sports sedentary group) and HF and LF/HF served as dependent variables, whilst controlling for VO_2_ max and BMI (Table 2). However, sport type had no significant effect on HRV parameters after controlling for VO_2_ max and BMI, *F* (20, 128) = 1.360, *p* = 0.155; Wilk’s *Λ* = 0.68 partial *η*^2^ = 0.175 (Figure 2).

Spearman correlation analysis demonstrated a modest association, ranging from low to moderate, between cognitive assessment variables, VO_2_ max, and measures of heart rate variability.

## Discussion

This study aimed to assess and compare heart rate variability and cognitive performance across various types of sports and compare these measures with a sedentary group. Based on existing literature, we hypothesized that regular sports training would be associated with improved cardiorespiratory fitness, heart rate variability outcomes, and cognitive abilities among open and closed skill sports players. The study’s findings supported this hypothesis, showing that individuals engaged in regular sports activities had higher cardiorespiratory fitness and better performance in sustained attention, working memory, and executive function than those who did not engage in regular sports activities. However, no significant differences in cognitive function or heart rate variability parameters existed between open and closed skill sport types.

### Cognitive performance and sport type

According to past research, physical activity positively impacts cognitive function [10]. Our study (Table 2) further supports these findings by indicating that individuals who engage in sports and have higher fitness levels tend to perform better in cognitive tasks [16,28]. Our study evaluated working memory using the EMT, and we found that both basketball players and swimmers exhibited faster response times, completed the task in fewer trials, and took less time than the control group. These findings align with previous research conducted by Moriya et al. who reported that engaging in moderate-intensity physical exercise was associated with increased activation of the right PFC and improved working memory performance compared to controls [29]. Our findings also revealed that basketball players and swimmers exhibited shorter completion times and fewer errors in the LCT, indicating improved sustained attention and concentration (Figure 1). This observation aligns with previous studies showing the positive impact of various physical exercises on enhancing focus and attention in individuals with attention-deficit disorders and intellectual disabilities [30,31]. Additionally, our results from the TMT indicated that basketball players and swimmers completed the test in less time than the sedentary group, consistent with previous research that has demonstrated a positive correlation between physical activity and executive functions [32]. Executive functioning involves regulating and controlling cognitive processes, and numerous studies have consistently shown a significant positive correlation between physical activity and enhanced executive functions [33,34].

In sports, inhibitory control plays a crucial role in successful performance. It refers to the ability to choose whether to execute or withhold an action within a short timeframe. We conducted a GNG and Stroop task to investigate inhibitory behaviour. Our findings indicated no significant difference in response accuracy to the GNG signals among the different sports groups (Table 2).

Numerous investigations have delved into the association between physical activity and cognition, and diverse theories have been proposed to unravel the biological mechanisms through which consistent physical training can boost cognitive functions. The study conducted by Colcombe et al. supported that individual with elevated levels of physical fitness demonstrated larger quantities of grey and white matter within their brains [35]. Erickson et al. similarly documented a positive correlation linking elevated fitness levels and augmented hippocampal volume, which in turn was connected with enhanced cognitive functioning [36]. Moreover, the comparative investigation conducted by Tseng et al. exhibited elevated levels of grey and white matter within the cuneus, precuneus, and subgyral areas of master athletes compared to sedentary individuals [37].

Our study found no statistically significant distinctions between the two exercise modes in various cognitive tasks. This echoes the study conducted by Guo et al. that the open skill sport type demonstrated better performances on visuospatial working memory than the sedentary control group. However, they found no differences between the open and closed skill sports groups [38]. An observational study conducted by Becker et al. also found that the two exercise modes were not significantly associated with executive function performance, including inhibitory control, working memory, and cognitive flexibility [39]. Furthermore, a systematic review by Gu et al. suggested that although most of the included studies supported the beneficial effects of the two modes of exercise on cognitive function compared with sedentary counterparts, evidence for superior cognitive function benefits of open sport type is relatively limited, due to a scarcity of long-term intervention studies [6].

However, it is important to note that basketball players performed better than swimmers across all aspects of the administered cognitive tests. Our findings align with the notion that athletes involved in open skill sports possess enhanced flexibility in attention, decision-making, and action execution compared to those engaged in closed skill sports [8]. This is consistent with previous studies conducted by Nakamoto et al. which have indicated that participants engaged in open skill sports, which impose high cognitive demands, demonstrate superior inhibitory control and executive functions [40]. The ability to efficiently process information in dynamic and unpredictable environments is a vital requirement for athletes in open skill sports, and it is closely linked to their superior cognitive performance and adaptability in decision-making and action execution [41].

### Sport type and HRV measures

In our investigation, we utilized a brief 5-min recording of ECG data to evaluate HRV. Elevated RMSSD, HF, and SD1 values were markers for increased parasympathetic control [42]. Our findings revealed that individuals participating in regular sports activities such as basketball and swimming displayed lower resting heart rates and higher RMSSD values, indicative of enhanced cardiac parasympathetic control. Our study revealed no significant differences between these two groups, as shown in Table 3.

Thayer et al. conducted research that proposed a correlation between a high resting-state HRV and the optimal functioning of prefrontal-subcortical inhibitory circuits, which are crucial for adaptive responses to environmental demands and executive functions [15,43]. Thayer et al.’s Neurovisceral Integration model suggests a connection between physiological vagal inhibition of brainstem acceleratory circuits and cognitive regulation [15]. Additionally, the brain regions associated with CAN described in the model have been linked to cognitive inhibition and conflict monitoring [44]. Based on these findings, we hypothesize that engaging in aerobic exercise training and participating in various sports activities may selectively activate this functional network, potentially enhancing inhibitory processes.

While our study reveals notable group differences in cognitive functions between athletes and non-athletes, we must acknowledge certain limitations in interpreting our results. Firstly, we did not directly measure the level of physical activity and the intensity of sports activities, which could influence HRV and fitness parameters in our study population. Additionally, the estimation of VO_2_ max was conducted indirectly. Despite efforts to maintain similar age and education levels across the three participant groups, there was an imbalance in gender distribution among them.

## Conclusion

The outcomes of this study align with prior research, reinforcing the notion that consistent participation in sports activities is linked to improved cognitive performance across various tasks when compared to individuals who do not regularly engage in sports. However, unlike previous studies that suggested better cognitive performance in open skill sports than closed skill sports, our results demonstrate no significant differences in cognitive task performance and heart rate variability measures between swimmers and basketball players. To gain a deeper understanding of the impact of these sports on cognitive performance and potentially uncover long-term improvements, future longitudinal studies should be considered.

### Ethical approval

The study was approved by Kasturba Medical College and Kasturba Hospital Institutional Ethics committee (IEC clearance: 811/2018) on 14 November 2018. This study was performed in line with the principles of the Declaration of Helsinki.

## Data Availability

Anonymized data are available upon reasonable request. Cognitive function test parameters in the three groups. LCT: letter cancellation task; TMT: trail making task; EMT: Ebbinghaus memory procedure test; GNG: Go/No Go task; *significantly different (*p* < 0.05) from sedentary group. Heart rate variability measures in the three participant groups. HR (bpm): resting heart rate in beats per minute (bpm); SDRR: standard deviation of all R-R interval; RMSSD: root mean square difference of successive R-R interval; HF: high frequency (HF; 0.15–0.4 Hz); LF: low frequency (LF; 0.04–0.15 Hz); SD1: standard descriptor 1; SD2: standard descriptor 2; nu: normalized unit; ms: millisecond; ms^2^: milliseconds squared. *Significantly different (*p* < 0.05) from sedentary group.
